# Increased cardiovascular risk in epilepsy

**DOI:** 10.3389/fneur.2024.1339276

**Published:** 2024-04-03

**Authors:** Mark L. Gaertner, Scott Mintzer, Christopher M. DeGiorgio

**Affiliations:** ^1^Department of Neurology, David Geffen-UCLA School of Medicine, Los Angeles, CA, United States; ^2^Olive View-UCLA Medical Center, Los Angeles, CA, United States; ^3^Department of Neurology, Thomas Jefferson University, Philadelphia, PA, United States

**Keywords:** epilepsy, sudden unexpected death in epilepsy (SUDEP), cardiovascular risk (CV risk), anti seizure drugs, epilepsy mortality

## Abstract

Epilepsy is associated with increased mortality. Cardiovascular disease confers a significant portion of this increased risk. Recently there is increased interest in the burden of cardiovascular mortality in people with epilepsy. This review discusses the most common cardiovascular risk factors and their association with epilepsy including obesity, diabetes mellitus, and hyperlipidemia. Hyperlipidemia related to the use of enzyme inducing anti-seizure medications is also discussed as a topic that is of particular importance to prescribers that have patients with comorbid cardiovascular risk and epilepsy. Heart rate variability (HRV) and its association with SUDEP is discussed as well as a contributor to vascular risk. Finally, the authors discuss a potential role for neurologists who treat epilepsy to engage closer with their patient's cardiovascular risk factors using available tools such as a the ASCVD score calculator to determine the overall risk of mortality, as well as acting upon this information to guide treatment approaches integrating the information provided in this review.

## Introduction

Epilepsy affects 3.47 million people in the US and 47 million people worldwide (1, 2). Mortality rates are higher in epilepsy than the general population. The standardized mortality rate for epilepsy vs. the general population is 1.74 (CI 1.64–1.87) (1). Epilepsy mortality rates are rising in the US (3). Age-adjusted mortality rates more than doubled from 5.6 per million in 2000 to 11.6 per million in 2017 (*p* = 0.001) (3). Causes for the increase in epilepsy mortality are multifactorial and include increased reporting, increased prevalence and higher than expected rates of cardiovascular disease (3, 4).

Cardiovascular disease and stroke are major causes of death in epilepsy. Stroke is the third leading cause of death and ischemic heart disease is the fourth leading cause (3, 4). Standardized incidence ratios for heart disease and stroke in epilepsy are higher than in the general population (Incidence Ratio 4.18 (CI 3.54–4.9) for heart disease and 4.96 for stroke (CI 4.19–5.84) (5). Risk for myocardial infarction (MI) is also higher in epilepsy compared to the general population (OR = 4.92, CI 2.34–10.31) and mortality after myocardial infarction is higher (Hazard Ratio 3.49, CI 1.05–11.65) (5, 6). The odds of death from stroke in people with epilepsy is 56% higher than in the general population (CI 1.27–1.98) (5).

In the US, stroke mortality in epilepsy increased from 6.3% in 2000 to 7.1% in 2018, an increase of 13.5% (*p* = 0.001) (4). This is counter to the 26% reduction in stroke mortality in the general population for the same period (4). From 2000 to 2018, mortality in the US from ischemic heart disease in people with epilepsy declined from 8.4% of all epilepsy deaths to 5.3%, a 34.4% reduction (*p* < 0.001), similar to the 39.7% decline in the US general population (OR 1.18 CI 0.94–1.47) (4). This confirms the rates of ischemic heart disease in epilepsy were declining at a similar rate to the general population (4).

However, there has been a recent reversal in this trend. Since 2015, the percentage of deaths due to ischemic heart disease among patients with epilepsy is now rising, counter to the trend in the general population (4). Although the causes are not fully elucidated, contributing factors for this trend reversal include increasing mortality in epilepsy due to hypertension, diabetes mellitus (to be referred to as diabetes for the remainder of the article), and stroke, which increased 82.5%, 70.4%, and 13.5% respectively since 2000 (4). This trend reversal is very concerning: cardiovascular mortality in people may be on the upswing after many years of declines.

## Vascular risk factors in epilepsy

People with epilepsy have higher overall risk for cardiovascular disease then the general population (7, 8). Bensken et al. reported the rate of hypertension was significantly higher in adults with epilepsy than the general population (8). Racial disparities in rates of hypertension in epilepsy are concerning (8). Black persons with epilepsy are at significantly higher risk for hypertension than white persons (52.7% in black persons vs. 33% in non-black persons) (8). Terman et al. (7) also reported a higher severity of cardiovascular risk factors in epilepsy. Using the U.S. National Health and Nutrition Examination Survey (NHANES) biannual survey to study vascular risk factors in the epilepsy population, Terman et al. (7) identified 154 individuals with epilepsy and 17,961 controls without epilepsy. Rates of hypertension, diabetes, and coronary heart disease were significantly higher in epilepsy than in those without epilepsy (7). Hypertension was present in 53% of adults with epilepsy, vs. only 39% in the general population (7). Diabetes affected 25% of epilepsy patients, vs. only 12% of those without epilepsy (7). This study by Terman et al. provides direct evidence that cardiovascular risk factors are more prevalent in those with epilepsy than those without.

Hypertension is also a risk factor for the development of late onset epilepsy (9). Vascular risk factors may account for up to 50% of late onset epilepsy (9). In a longitudinal study of a Framingham cohort of 2,986 subjects ages 45 and older, new onset epilepsy occurred in 55 subjects (9). The presence of hypertension conferred a significant increase in risk for developing new onset epilepsy (Hazard ratio 1.93 (CI 1.1–3.37, *p* = 0.02) (9).

## Obesity

Obesity, a principal risk for Type 2 diabetes, is a major problem in epilepsy (10). In a cohort of 554 consecutive new patients with epilepsy ages 16–87, 55.2% were overweight (BMI > 25 kg/m^2^), and 31.2% were obese (BMI > 30 kg/m^2^) (10). Likely contributors include drug effects, sedentary lifestyle, and coexisting intellectual disability (10). Treatment with multiple anti-seizure medications (ASMs) is more common in obese patients with epilepsy than in those of normal weight (37.7% vs. 25%) (10). A direct cause-and-effect relationship between the number of anti-seizure medications and obesity remains unclear, but obesity itself is associated with an increased risk of drug resistant epilepsy. 36.9% of those with drug resistant epilepsy are obese compared to 24.6% with well controlled epilepsy (10). This may result from higher numbers of ASM's in drug resistant epilepsy than well controlled epilepsy, as well as reductions in physical activity.

Intellectual disability, autism, and developmental disorders contribute to obesity and elevated cardiovascular risk in epilepsy. Persons with intellectual disability have a higher overall prevalence of epilepsy, in one systematic review the rates ranged from 9–51.8% (11). Rates of obesity are also significantly higher in those with intellectual disabilities (12). The combination of higher rates of intellectual disability and comorbid obesity likely are factors contributing to the higher risk for cardiovascular risk in epilepsy.

## Diabetes

People with epilepsy are at higher risk for diabetes. Of 154 persons with epilepsy in the NHANES cohort reported by Terman et al., 25% had co-morbid diabetes, vs. only 12% of those without epilepsy (difference *p* < 0.01) (7). Diabetes is widely known to increase the risk of cardiovascular disease and stroke. The spectrum of risks of diabetes to people with epilepsy has been better defined. In a Taiwanese hospital cohort of 92,438 people with diabetes, epilepsy conferred higher hospital mortality rates and worse outcomes (13). Thirty-day hospital mortality was significantly higher in people with diabetes and epilepsy when compared with diabetes without epilepsy (1.8% vs. 1.1%, CI 0.98–2.60) (13). The rates of sepsis and pneumonia were also significantly higher in epilepsy with co-morbid diabetes than diabetes alone (13). Wilner et al. evaluated health care claims in patients with epilepsy and found an increasing prevalence rate of diabetes with advancing age (14). Rates of diabetes in individuals ages 19–64 years old was 7% and increased to 14% in those over 65 years old (14). Arend et al. compared biomarkers for diabetes and metabolic syndrome in people with epilepsy and found that fasting glucose levels were elevated in people with epilepsy vs. controls (93.76 mg/dl sd 2.87 vs. 84.85 mg/dl sd 1.41, *p* < 0.05) (15). People with epilepsy are at higher risk for diabetes than the general population.

## Hyperlipidemia

Hyperlipidemia is also a major co-morbidity in people with epilepsy (16). In a population-based study in Taiwan, Harnod et al. found a significant increase in risk for hyperlipidemia vs. the general population (16). Of 900 people with epilepsy, the incidence of hyperlipidemia was 34.1 per 1,000 person-years, vs. only 26.9 in the general population (incidence ratio of 1.28), for an adjusted hazard ratio of 1.17 (16). The highest risk was in people ages 50–59, where the hazard ratio was 1.35 (16). An observational cross-sectional study using 110 patients with epilepsy and 46 age matched controls demonstrated that the prevalence of abnormally elevated non-high-density cholesterol (i.e., LDL and VLDL) was higher in the cohort with epilepsy than in controls with a frequency of 51% vs. 30.4% (17). Similarly, Arend et al. reported lipid levels in a case-control study comparing 32 persons with epilepsy to 41 controls (15). Low-density lipoprotein levels were significantly elevated compare with those without epilepsy (129 SD 8.45, vs. 76 SD 2.02, *p* < 0.0001) (15). Wilner et al. in their large cohort of 6621 persons with epilepsy, found that prevalence rates of hyperlipidemia were 15% in those ages 19–64, and 29% in those over 65 years old (14). Similar to diabetes, hyperlipidemia is more common in people with epilepsy than the general population and the rates increase with advancing age.

## ACC-ASCVD risk in epilepsy

The ACC-ASCVD score is a tool that integrates age, sex, systolic blood pressure (SBP), diastolic blood pressure (DBP), smoking, diabetes, high density lipoprotein (HDL), and low-density lipoprotein (LDL) into a single 10-year risk score for cardiovascular disease and stroke (18, 19). The current tool is based on a pooled cohort equation, which integrated data from four large US studies, including the Atherosclerosis Risk in Communities study, the Cardiovascular Health Study, the Coronary Artery Risk Development in Young Adults study, and the Framingham Heart Study (REF) (18, 19). Terman et al. reported ASCVD scores in all people with epilepsy enrolled in the NHANES Survey for the period 2013–2018 (7). The 10-year ASCVD score for 127 subjects with epilepsy was higher than the 15,493 without epilepsy (6.1% SD 8.2% vs. 5.2% SD 8.3%, *p* = 0.048) (7). This provides evidence that when integrating age, gender, smoking history, blood pressure and lipids, people with epilepsy regardless of insurance status have elevated 10-year risk for heart disease and stroke compared with those without epilepsy.

## The role of anti-seizure medications on lipids and vascular risk

Treatment with anti-seizure medications likely contributes to the increased risk of hyperlipidemia and cardiovascular risk in people with epilepsy (20–23). Cytochrome P450 enzyme-inducing anti-seizure medications may play an important role in increasing cardiovascular risk. Mintzer et al. conducted a crossover study in which patients with epilepsy on cytochrome P450 enzyme-inducing anti-seizure medications (e.g., phenytoin or carbamazepine) were converted to non-enzyme inducing anti-seizure medications, (Lamotrigine or levetiracetam) (21). Total cholesterol, LDL, homocysteine, and C-reactive protein were measured at baseline and 6-weeks after the conversion, and the change at 6-weeks was compared to 16 normal controls (21). Six weeks after conversion, total cholesterol declined 24.8 mg/dl, LDL declined 19.9 mg/dL, and triglycerides declined 47 mg/dL (21). C-reactive protein, an inflammatory marker strongly associated with risk for stroke and heart disease, declined 31.4%, *p* < 0.03 (21). Similar results were obtained when patients were switched from carbamazepine or phenytoin to topiramate, lamotrigine, levetiracetam, or zonisamide (23). An earlier study by Isojarvi et al. found that switching from carbamazepine to oxcarbazepine also results in reduction in serum lipids (24). These results affirm that the cytochrome P450 induction properties of these drugs are likely responsible for their hyperlipidemic effects. One question that arises is whether these numerical changes are of clinical relevance. To address this, Mintzer et al. used the Market Scan Commercial and Medicare databases to perform a population-based study to evaluate prescription data from 11,374 newly treated persons with epilepsy (21). Treatment with enzyme inducing agents was associated with a >22% higher odds of a subsequent clinical diagnosis of hyperlipidemia (OR 1.225, CI 1.066–1.408) (21). Mechanisms for why common enzyme inducing ASMs elevate plasma lipids and other cardiovascular biomarkers remain to be clarified, though it has been hypothesized that induction of CYP51A1, which plays a crucial role in the cholesterol synthetic pathway, may be at least partly responsible (25).

There have been additional lines of evidence validating the clinical importance of these effects. A Taiwanese study demonstrated that duration of therapy with the enzyme inducers phenytoin and carbamazepine, but not lamotrigine, correlated with increased thickness of the carotid intimal-medial layer on ultrasound; the latter is a very specific marker for atherosclerosis and vascular risk (26). This study validates the notion that increased vascular risk in patients taking these drugs is indeed likely due to accelerated atherosclerosis rather than some other mechanism (26). The study also found that valproate, an enzyme inhibitor, similarly increased carotid thickness; it remains unclear what might underlie this (26). Josephson et al., using National Health Service data from the United Kingdom, evaluated the long-term risk of cardiovascular disease in a cohort of 50,000 persons with epilepsy. Enzyme inducing anti-seizure medication exposure was associated with a net 21% increase in the incidence of vascular disease (27). However, Lee-Lane et al. also performed a retrospective case control study In Wales and found that exposure to enzyme inducing anti-seizure medications was not associated with an increase in cardiovascular events compared with those exposed to non-enzyme inducing anti-seizure medications (28). Further work is necessary to confirm that enzyme inducing anti-seizure medications increase the risk of endpoint clinical vascular disease.

## Heart rate variability and vascular risk

Heart rate variability (HRV) is the beat to beat variation of the heart rate, usually calculated from R-R intervals as recorded during continuous cardiac monitoring using Holter monitors, implantable loop recorders or wearable devices (29). The central nervous system (CNS) regulates the heart rate and its variability in response to sleep or awake state, respiration, exercise and stress (29–32). HRV can be expressed using time-dependent methods (SDANN, SDNN, and RMSSD) or frequency-dependent methods using fast Fourier transform algorithms (e.g., Low Frequency Power (LFP) The vagus nerve acts to reduce the heart rate via the SA and AV nodes, and acts to protect the heart during hypoxia, ischemia and stress. Reduced heart rate variability (especially reduced LFP and RMSSD) are associated with the risk of cardiac mortality from arrythmias and sudden cardiac death (33, 34). Similarly, reduced LFP and RMSSD measures (specifically low awake RMSSD values and high Sleep/Wake RMSSD ratios) are associated with the risk of SUDEP (31, 32, 35, 36). The exact mechanisms linking HRV alterations to SUDEP remain an active area of investigation, but the correlation between HRV, especially LFP and RMSSD underscores the importance of monitoring HRV as a biomarker for SUDEP risk (31, 32, 35, 36). From the data, it is evident that abnormal values of LFP and RMSSD in epilepsy patients indicates autonomic dysfunction which is linked to increased risk of SUDEP (31, 32, 35, 36). This highlights HRV's potential as a biomarker for SUDEP risk assessment and calls for further research into epilepsy's autonomic and cardiac effects to mitigate SUDEP risk.

## Potential role of neurologists in cardiovascular risk surveillance and treatment

Since neurologists primarily focus on diagnosis, prevention and treatment of seizures, surveillance for modifiable cardiac risk factors in neurology clinics may be ignored or deferred. The problem of neurologists not routinely monitoring or treating cardiovascular risk factors is now getting greater attention (37–39). As stated in a recent editorial commenting on the failure of neurologists to treat blood pressure, “[*Neurologists] dropped the hammer on hypertension 20 years ago!*” (37). Limited surveillance and treatment of cardiovascular risk factors by neurologists may result in missed opportunities to prevent heart disease, diabetes, and stroke. The evidence indicates that when Neurologists do treat hypertension, outcomes are improved (38). There may be a role for neurologists to intervene in initiating treatment for uncomplicated hypertension and hyperlipidemia, but this will require a significant shift in awareness and practice (39). More than anything, it will require increasing coordination and communication with primary care practitioners and cardiologists, particularly regarding drug treatment (39). Physicians in other specialties rely on neurologists to optimize the patient's epilepsy treatment in all respects, not merely the prevention of seizures. Just as a neurologist would know that depression or anxiety can be worsened by levetiracetam, resulting in a change in therapy, it is incumbent upon neurologists to understand that treatment with enzyme inducing antiseizure medications may heighten vascular risk and act accordingly, because internists and cardiologists will almost certainly not dare to make alterations in seizure medications. In this way neurologists may contribute to improving patients' cardiovascular health and reduce the risk of stroke and heart disease in people with epilepsy (39).

## Limitations

The scope of this mini-review does not allow an exhaustive systematic review of all relevant literature but is focused on key highly cited articles selected by the authors. The articles were chosen to highlight key associations between epilepsy and cardiovascular risk factors. A more comprehensive and systematic review is warranted to better characterize these associations with broader scope and detail.

## Author contributions

MG: Conceptualization, Validation, Writing – review & editing. SM: Conceptualization, Writing – review & editing. CD: Conceptualization, Writing – review & editing, Writing – original draft.
